# On the Vibration of the Iris, the Spontaneous Passage of the Crystaline Humour into the Anterior Chamber of the Eye, and Its Restoration, by the Application of Extract of Bella-Dona

**Published:** 1809-12

**Authors:** M. Becquet


[We profess ourselves not sufficiently masters of the subject contained in the following paper. It was, however, thought right to extract it from the French Journal, that our countrymen may favour us with their animadversions; and, if the subject is generally new, that they may direct their attention to ascertaining the reality of the fact.]

### *On the Vibration of the Iris, the spontaneous Passage of the crystaline Humour into the anterior Chamber of the Eye, and its Restoration, by the Application of Extract of Bella-dona*; by M. BECQUET.*

IT is not at present my intention to describe diseases of a new character: it is merely my wish to detail facts, which

* Journal de Medicine.

which are met with but rarely in practice, and with which it is important to be acquainted. Although those I am about to record are not precisely identical, they have nevertheless sufficient analogy to entitle them to a place in the same memoir.

The vibration of the iris is the state in which it is agitated by more or less rapid undulations backward and forward. These motions are merely passive, since they are only perceptible when the eyes are moved by their muscles.

Although this phenomenon is not of frequent occurrence, I have met with it in several cases. It generally appears without being preceded by any peculiar affection; it is unattended with pain, and usually ends with loss of sight before the age of 35. So far at least as my experience goes, this affection has never been preceded by any peculiar sensation, nor accompanied by pain. It commences in infancy, and continues during life:* the sight is very weak, and so short, that those affected in this way cannot read, except with concave glasses, of a focus from two and a half to three inches. All the adults affected with this malady bear testimony, that in their infancy their sight was very weak, and excessively short; some inform us that they have experienced a kind of dimness, or sudden and momentary loss of sight.

The vibration may exist with or without opacity of the crystaline humour, and in one or both eyes. During the infancy of those who are affected in this way, the pupil is large and regular; the iris is susceptible of constriction or dilatation, even in a considerable degree, according to the modification of the light. In adults and old persons the pupil is contracted, frequently deformed, and sometimes accompanied with opacity in the crystaline humour; the latter always seemed to me to be diminished in volume, and of a glary colour, as well when it retained its orbit, as when it had deviated from it, either to one side or downwards.

Case I. Upwards of thirty years ago I saw a person, of the age of 60, affected with this disease; the vibration of the iris existed in one eye, which, although there was an almost total obscurity of vision, was in appearance perfectly sound; the patient ascribed his loss of sight in this eye to a *coup de sabre* received in battle, on the superciliary ridge,

* I have scarcely ever observed it before the age of seven.

ridge, which exhibited a long cicatrix. The other eye was recently affected with cataract, which had come on gradually. The operation for the cataract was performed in my presence, by the late Grand-Jean; but restoration of sight was not effected, for the pupil became much contracted, and was obscured by some remains of an opaque substance. The patient continued, however, to distinguish night from day with as much accuracy as before the operation.

This case proves, that notwithstanding the vicious state of one eye, a state resulting from the vibration of the iris, the operation for the cataract may be performed on the other eye with success. In fact, if the cataract had been of an older date, and had acquired more solidity, neither the humour of Morgagni, nor the membranous part which became opaque, would have remained after the operation.

Case II. I saw an American of about 11 years of age, one of whose eyes was affected with a vibration of the iris, and with cataract at the same time. According to the account of the parents, the child had lost the sight of that eye three years ago: the other eye was perfectly sound.

Cases III. IV. and V. I am acquainted with three other persons, two brothers and one sister, who have been affected for about twelve years with the vibration of the iris, attended with peculiar circumstances.

These cases have supplied me with the chief materials for the present memoir; and the history of their disease is so curious and interesting, that I think it merits attention:—

In 1795, I was requested to visit the eldest of these three children, then about 12 years of age. I was informed that, two months before, this young man having been accidentally in the prisons of Arras, and being desirous of sawing a piece of timber, placed his foot on one of the shoulders of the saw, and bent his head violently forward: he instantly experienced a dimness in the right eye, accompanied by a decreased sensibility. The physicians and surgeons of Arras were consulted, and being totally unacquainted with the disease, advised the parents to take the child to Paris. All the oculists of the capital were successively consulted, and all of them ascertained, that the crystaline humour had passed into the anterior cavity, where it appeared behind the cornea like a round spot, four lines in diameter.

All the medical attendants, taking into consideration the the age, indocility, and untowardness of the patient, the repugnance of the parents to an operation of doubtful issue, and finally, the absence of pain from the diseased eye, concurred in recommending, that it should be left to nature, with the exception of merely watching the future progress of the disease. M. Winsel alone dissented from this advice, and suggested, that an operation should be instantly performed. His opinion, however, did not prevail; but the event proved that he was right.

The young man returned to Arras, and the eye continued in the same state during four months. About this period he experienced a most serious and obstinate attack of ophthalmia, which at last terminated by the spontaneous opening of the cornea, and by the issue of the crystaline humour throughout the aperture.

Although I had recommended the eye to be kept always completely closed by a bandage after the crystaline humour had come away, this precaution was neglected; and the cornea increased so much in size, that the eyelids could scarcely cover it.

In the month of May, in the year following, the young man came to Paris, when I found him in the state described; the eye, however, was a little red and sensible. I recommended that it should be kept shut, but not with a hard compress ,as hitherto used. My intention was to moderate the inflammation, to support the cornea in order to diminish its projection, and to allay the pain, which were effected in the space of six weeks.

It was on this occasion that I first saw the sister of this young man. She had in both eyes a very apparent vibration of the iris; her eyes were large, and she was very short-sighted. I made her read with concave glasses, as well as her two brothers, in whom I observed no vibration of the iris, either from its being weak and not frequent, or from its not yet existing. I gave some directions which were but imperfectly attended to, and the children returned to Arras.

The year following I was informed that the young lady had entirely lost the sight in one eye: she was brought to Paris, and I attended her without success. Eighteen months afterwards she gradually lost the use of the other eye in a similar manner.

I shall now resume the history of her brother's case. In 1803, he was brought to Paris; his eye was by no means painful, although the cornea was very much dilated, and he he found it answered very well to cover it with a black ribbon.

In 1804, in the month of May, the whole family returned to Paris. I examined the eyes of the youngest of the two brothers, in whom I perceived a very apparent vibration of the iris in both eyes; I prescribed an appropriate regimen, and applied a seton, which the patient kept up. The elder brother, being anxious to get rid of his bandage, and yet being unable to bear the impression of the air and light, requested me to amputate the superabundant part of the cornea. I yielded to his entreaties, persuaded as I was that the superabundance produced iritation.

The amputation was performed in an instant, and a hæmorrhage came on, but it was of short continuance. The patient was seized with a vomiting, which lasted ten or twelve hours. The consequent discharge from the eye diminished so rapidly, that, on the second day after the operation, the young man was able to wear a glass eye; this was laid aside, however, for three or four months, when he had occasion to take a journey.

Some time afterwards, on examining if the glass eye was a good resemblance of the eye which I thought sound, I was very much astonished to perceive in the latter a feeble vibration of the iris; my astonishment arose from having so frequently seen this young man, without having remarked this circumstance. It is, however, possible that this vibration may have previously existed, only in a very feeble manner; likewise, when I examined the protuberant eye, performing the operation, I conducted the patient into a dark corner, for he had always the precaution to conceal the other eye with a handkerchief; so that it was extremely difficult to remark this vibration, if it existed. It is even possible that the affection did not manifest itself until the period of the operation on the other eye, or some time afterwards.

I confess I began to regret that I had amputated the cornea in order to substitute a glass eye, and I was fearful that the presence of this foreign body would be injurious to the other eye. I was even inclined to make a seton in the neck; but hesitated, from the reflection that the glass eye would serve as a seton; in fact, we may almost compare a glass eye to a large issue-pea; like the latter, it may occasion a daily and abundant exudation.

This young man came to see me in the month of May, 1806; he told me, that for a long time he had experienced a kind a kind of dimness, of which he had always forgotten to inform me; that his younger brother had also been subject to the same sensation; that he had regarded it as a thing which was natural; that it took place involuntarily several times a day, and that it occurred whenever the head was bent towards the ground in order to lift up any thing, on which occasion they could discern nothing. He then proceeded to give me a proof of it; and having gone to an obscure corner of the room in which we were, he bent his head downward, and informed me that the dimness had taken place; on his approaching the window I observed, to my great astonishment, the same phenomena which took place in the other eye in the prisons at Arras. The crystaline lens placed in the anterior chamber was completely transparent, and resembled a large drop of very fine oil. The young man, according to the habit he had acquired, immediately put the crystaline back into its place, by covering the eye with his hands, and throwing his head back. I warned him against exposing himself in future to similar experiments, lest he should one day fail in reducing the lens.

This experiment took place in presence of several persons who were in the apartment.

The two brothers having visited me in May, 1807, the eldest informed me, that since he had followed my advice, he had experienced scarcely any dislodgment of the crystaline, whereas his younger brother, who did not follow the same precautions, was affected in this manner three or four times a week.

I repeated the recommendations I had already given, to avoid bending his head downwards, particularly in a dark place; because their pupils being susceptible of great dilatation, it might happen, that the crystaline lens could not return, and it might be necessary to extract it, experience having shewn that its continuance behind the cornea was dangerous in the extreme. I also informed them, that if this circumstance took place, the reduction of the humour could not be effected in less than two or three hours; and that it became requisite to facilitate the dilatation of the pupil, and consequently, the reduction of the crystaline humour, by the application of the extract of bella-donna. I described the method of using it at the same time.

Case VI. On the 16th of May, 1807, a tailor, aged 60 came to consult me on behalf of a sick friend. When conversing with him, I perceived that he was affected with a vibration of the iris in the left eye. To the questions which I put to him, he answered that he was always short sighted, that his sight had gradually failed, and that at the age of 27, he had suddenly lost the use of the left eye when drinking his share of a bottle of wine.

On a closer examination, I discovered that this vibration of the iris was rapid, and perfectly in unison with the smaller motions of the eye. The pupil was a little contracted, transversly deformed, elliptic, sunk, and without any motion of dilatation and contraction in different shades of light. Beyond the pupil I perceived a small white spot, which I thought was the crystaline lens naturally depressed. In order to assure myself of this, I placed the patient on his knees, stooping towards the ground, and in this state shook his head violently. When he rose, I saw the crystaline lens almost entire, a little smaller than in the natural state, of a glary colour, and exceeding two-thirds of the pupil.

After this examination, it occurred to me, to place a little extract of bella-donna on the cornea, to ascertain if under these circumstances, the extract acted in the iris; but as I was fearful of doing an injury, I did not make the attempt.

I could mention a variety of analogous cases, which have occurred during a practice of thirty years, particularly when I have been applied to by short sighted persons; but as my memory does not supply all the particulars, I have confined myself to the foregoing.

*Reflections.*—I conceive myself to be the first, who in cases of vibrations of the iris, has remarked the spontaneous and reiterated passage of the crystaline lens into the anterior cavity. The vibration of the iris itself is very little known; and authors mention it in a vague manner only. It is, however, perceptible to an observer, and frequently serious enough to produce loss of sight.

What is the cause of the vibration of the iris, of the detachment of the crystaline from the coat of the vitreous humour, and of the loss of sight which generally ensues with it, without opacity of the crystaline humour? I confess, that on this head I can only found some conjectures on mere probabilities. If we refer to books, very few authors have mentioned the subject, and those who have done so, are Maitre Jean, Messrs Winsel and Scarpa. It would seem that Maitre Jean saw the vibration of the iris in conjunction with the fluctuating cataract; he ascribes the cause of it to the solution and diminution of the vitreous ous humour: in his chapter on the fluctuating cataract, p. 219, he says: "The following are the symptoms of this disease: When the solution is effected by a deposition of humour, the patients at first complain that they see very little, or not at all, although no change is remarked in the eye, with the exception of the pupil being a little more dilated than ordinary, and this loss or diminution of sight is sometimes preceded by violent pains in the head nd at the bottom of the eye, while, at other times, the natients are free from pain: a short time afterwards, pthe crystaline is seen very turbid, and latterly becomes white, then yellow, and on the least motion of the eye it is seen vibrating like a vane in a light wind; the iris losing its natural colour, becoming shrivelled, and moving sometimes backward and forward, according as it is agitated by this floating crystaline."

It should seem from the above quotation, that Maitre Jean only saw the vibration of the iris in the case of the fluctuating cataract, or never saw it until it had been of several years standing.

I have seen few vibrating cataracts for a long time past, and in such cases as I have seen, I do not recollect to have remarked any particular motion in the iris. Glaise seems to have observed such cataracts, which he ascribes to blows or falls, and does not appear to have observed any motion of the iris; at least, he has not mentioned any. In his chapter on the external causes of cataracts, p. 12, he says. "It is proper to remark, that when the fall or blow has been very violent, the crystaline lens is partly displaced from its capsule, begins to vaccilate, and at length produces a cataract called *branlante*. This cataract is incurable, and is always accompanied with gutta serena."

M. Winsel adds, by way of note to his 28th case, p. 139, edition of 1786. "The phenomenon of the oscillating motion of the iris, to which oculists do not seem to have paid much attention, happens frequently after the operation for the cataract; it is very difficult to describe, although it is easy to perceive it; it is a kind of undulation, which seems to be produced by the aqueous humour, although this humour does not undergo a real displacement. The cause of this singular motion, (and it is independent of the contraction and dilatation of this membrane) seems to be owing, in a great measure, to the absence of the crystaline humour, and the iris being consequently less supported.

Such was the opinion of M. Winsel twenty-one years

 ago; ago: I am persuaded that he is now of a different opinion. No oscillatory motion of the eye exists in those who have undergone an operation for cataract, and there is reason to believe, that this motion depends on another cause.

Scarpa has remarked the tremulous motion of the iris, and ascribes it to dropsy in the eye: he says, p. 217, Vol. II. 1st edition, speaking of falls or blows. "At other times, this disease is not the effect of any of these or other manifest causes, particularly if it occurs in children of a very tender age, from whom we can learn nothing. The eye has no sooner assumed the oval figure, and the anterior cavity has no sooner assumed an extraordinary capacity, than the iris seems to be placed more forward than usual, and is singularly tremulous on the smallest motion of the globe of the eye; the pupil continues dilated in every shade of light, and the crystaline humour is brown from the beginning of the disease, or it is not obscured until the affection has attained a greater height. The disease then becomes stationary; the crystaline lens is not very opaque: the patient distinguishes light from darkness, can see a little of the outline of a body, and can distinguish the most striking colours. But does the eye become enlarged? Is the whole crystaline lens bedimmed? At length the retina becomes palsied, by the excess of distension, and consequently, is not sensible to the few luminous rays which reach the bottom of the eye, after passing the edges of the crystaline humour."

In several cases of oscillation of the iris, I have observed, that the eyes were bulky; in others, they were smaller than usual; while in other cases, they were of the common size. What conclusion can be drawn from these different states? The cause must be sought for elsewhere.—When we consider that this disease does not occur in infancy, there are grounds for ascribing it to a vicious organization; this chiefly exists in the vitreous humour, which is dissolved, or at least has not its ordinary consistency, is less in quantity, and no longer adheres to the crystaline lens, or its capsule, if there are not some vessels forming a kind of peduncle, and serving to carry nutrition to it. Hence it follows, that there is no collet or boazel to the vitreous humour: that this humour is almost spherical, and that the crystaline touches the iris. Hence it follows also, that the vitreous humour, and the crystaline lens, being agitated during the motions of the eye, impress on the iris the tremulous motion observed.

It is to be remarked, that in the early stages of this disease, ease, no separation takes place between the ciliary ligament and the cemented passage. Until this separation is established, the sight, however bad, never entirely fails. At length, however, the crystaline lens presses continually on this union, and insensibly detaches the ciliary ligament; the crystaline then quits the vitreous humour, is precipitated either vertically or sideways, between the retina and the vitreous humour. The retina is injured; the sight is lost; the crystaline lens becomes opaque, and is diminished in volume.

All this is founded on probabilities only; for in order to be certain that these things happen, the operations of nature must be traced by dissections, which can only be practised in Hospitals, where a greater number of diseased cases are to be found.

It is perhaps, easy to explain why those who are afflicted with this disease are extremely short sighted. Admitting, as I have alleged, that the vitreous humour is totally spherical, that the setting of the crystaline lens does not exist any longer, and also, that the crystaline humour is far behind the pupil, the crossing of the rays is already diminished to such a degree, that extremely concave glasses are requisite for lengthening the crossing or focus: if in this state, the crystaline quits the visual axis, it produces dimness, and if the pupil be susceptible of great dilatation, the crystaline humour passes into the anterior chamber, the dimness is then carried to such a pitch that the patient can no longer distinguish objects.

We may now ask, why does the oscillation of the iris manifest itself only on infants and young people, and continue during life? What is the cause of the supposed solution of the vitreous humour, and what are the means of remedying it? How does it happen, that in some persons the opacity of the crystaline humour is early declared, in others later, and in others not at all? or at least, wherefore is it not apparent? Why do some persons lose their sight at an early age, and others preserve it until the age of thirty and upwards? How is the crystaline humour enabled to pass into the anterior chamber, and be replaced several times a day? Why, in short, does it not lose its transparency?

Some of these difficulties seem to have been resolved, but there are others which I am under the necessity of leaving to the examination of other observers.

The following practical hints may be gathered from the foregoing pages. 1st. That in the case of the displacement, or spontaneous passage of the crystaline into the anterior chamber, from the head being bent downward in a dark place, we may, by a contrary situation, in the dark, or by keeping the eyes quite close, and by some motion of the head, replace the crystaline humour in the posterior chamber, and thus restore the sight, if it had not been lost before the accident.

2d. That the crystaline humour, when passed into the anterior chamber, cannot remain there several months without occasioning inflammations, and a suppuration from the eye, and consequently, the necessity of a glass eye, if the crystaline humour be not speedily replaced in the posterior chamber.

3d. That the dimness which characterizes the passage of the crystaline humour into the anterior chamber, and which is always followed by the loss of sight, is dissipated by its reduction.

4th. That all delays in attempting this reduction are dangerous.

5th. That if the reduction of the crystaline humour in the posterior chamber, could not be produced by the means pointed out, we must facilitate it by producing a dilatation of the pupil with extract of bella-donna.

I shall conclude by pointing out the method of applying this remedy.

A small camel's hair pencil dipped into the bottle, which contains extract of bella-donna, a little diluted in water, in order to touch the surface of the cornea, seems to me to be best adapted for this purpose, and it is proper to repeat the operation twice or thrice in the space of twenty minutes. For as the dilatation of the pupil does not take place all at once, I do not think that a single application would suffice. When this dilatation is obtained, the patient throwing up his head with his eyelids shut, in a dark place, a few shakes of his head will be sufficient to reduce the crystaline lens.

It is essential to observe, that as the pupil requires eight days to resume its natural state, it is necessary that the patient should avoid darkness, and never throw down his head during this period. On the contrary, he ought to lie on his back, exposed to a strong light.

Cases

